# Effect of a package of integrated demand- and supply-side interventions on facility delivery rates in rural Bangladesh: Implications for large-scale programs

**DOI:** 10.1371/journal.pone.0186182

**Published:** 2017-10-26

**Authors:** Sayedur Rahman, Aziz Ahmed Choudhury, Rasheda Khanam, Syed Mamun Ibne Moin, Salahuddin Ahmed, Nazma Begum, Nurun Naher Shoma, Md Abdul Quaiyum, Abdullah H. Baqui, for the Projahnmo Study Group in Bangladesh

**Affiliations:** 1 Johns Hopkins University-Bangladesh, Dhaka, Bangladesh; 2 International Center for Maternal and Newborn Health, Department of International Health, Johns Hopkins Bloomberg School of Public Health, Baltimore, MD, United States of America; 3 International Centre for Diarrhoeal Disease Research, Bangladesh (ICDDR,B), Dhaka, Bangladesh; National Academy of Medical Sciences, NEPAL

## Abstract

**Background:**

According to the Bangladesh Demographic and Health Survey 2014, only approximately 37 percent of women deliver in a health facility. Among the eight administrative divisions of Bangladesh, the facility delivery rate is lowest in the Sylhet division (22.6 percent) where we assessed the effect of integrated supply- and demand-side interventions on the facility-based delivery rate.

**Methods:**

Population-based cohort data of pregnant women from an ongoing maternal and newborn health improvement study being conducted in a population of ~120,000 in Sylhet district were used. The study required collection and processing of biological samples immediately after delivery. Therefore, the project assembled various strategies to increase institutional delivery rates. The supply-side intervention included capacity expansion of the health facilities through service provider refresher training, 24/7 service coverage, additions of drugs and supplies, and incentives to the providers. The demand-side component involved financial incentives to cover expenses, a provision of emergency transport, and referral support to a tertiary-level hospital. We conducted a before-and-after observational study to assess the impact of the intervention in a total of 1,861 deliveries between December 2014 and November 2016.

**Results:**

Overall, implementation of the intervention package was associated with 52.6 percentage point increase in the proportions of facility-based deliveries from a baseline rate of 25.0 percent to 77.6 percent in 24 months. We observed lower rates of institutional deliveries when only supply-side interventions were implemented. The proportion rose to 47.1 percent and continued increasing when the project emphasized addressing the financial barriers to accessing obstetric care in a health facility.

**Conclusions:**

An integrated supply- and demand-side intervention was associated with a substantial increase in institutional delivery. The package can be tailored to identify which combination of interventions may produce the optimum result and be scaled. Rigorous implementation research studies are needed to draw confident conclusions and to provide information about the costs, feasibility for scale-up and sustainability.

## Introduction

The maternal mortality ratio (MMR) in Bangladesh has substantially decreased from 322 deaths per 100,000 livebirths in 1998–2001 to 194 deaths per 100,000 livebirths in 2007–10 [1]. The neonatal mortality rate also declined from 41 per 1,000 livebirths in 1999–2003 to 28 per 1,000 livebirths in 2010–14 [2]. Despite this progress, the numbers of maternal and neonatal deaths remained high. Delivering at health facilities can prevent many of these unnecessary deaths [3–7]. Nonetheless, utilization of health facilities for delivery care, which is a proxy for use of skilled obstetric care, is still quite low in Bangladesh [2]. In Bangladesh, the proportion of mothers delivering in health facilities has been persistently low (29 percent in 2011 and 37 percent in 2014), but the rates are even lower in rural areas (31 percent in 2014) among the poorest quintile of the population (15 percent in 2014) and in Sylhet (23 percent in 2014), which is one of the eight administrative divisions in Bangladesh [2]. The maternal and neonatal mortality rates are higher in these sub-populations. In 2010, compared to the national average MMR of 194 per 100,000 livebirths, the MMR was 199 per 100,000 livebirths in rural populations, 234 per 100,000 livebirths in the poorest quintile and 425 per 100,000 livebirths in the Sylhet division [1]. The neonatal mortality rate in the year 2014 was 31 per 1,000 livebirths in rural populations, 35 per 1,000 livebirths in the lowest wealth quintile and 39 per 1,000 livebirths in the Sylhet division [2].

There are several factors in the healthcare system that may influence a woman's ability to access appropriate care during pregnancy and child birth. Thaddeus and Maine [8] developed a three delays model about two decades ago to evaluate the circumstances surrounding access to skilled attendance at delivery. The authors defined the factors, chronologically, as the lengths of the delays in (i) the decision to access care, (ii) reaching an adequate health care facility, and (iii) the receipt of adequate and appropriate care. Distance to health facilities, lack of transport, inadequate financial resources to access care, and poor-quality care, which are often interlinked, are fairly common problems in resource-poor settings such as Bangladesh [9–13]. Women, particularly in rural areas, often prefer to deliver at home rather than embarking on a long and difficult journey to poorly attuned public health facilities that often suffer from shortages of supplies, equipment and trained personnel, leading to poor quality of care [14–17]. In many public health facilities, patients have to pay out-of-pocket for everything, including unofficial user fees [18–22]. While delivery is the single most costly event during pregnancy, the cost of complicated deliveries is often catastrophic, which can force poor households into deeper economic hardship and poverty [23, 24]. Furthermore, many social factors may influence the decision to seek care, such as lack of awareness of the value of maternal health services, lack of information of sources of care, cultural norms and beliefs system, traditional practices that include a preference for birthing at home, and requiring permission from family decision makers [9, 14, 25].

Given the complexity of these barriers, it is suggested that interventions aiming at improving maternal health outcomes need to be multi-pronged and comprehensive so that they explicitly address both health service constraints on the supply side as well as contextual factors, for example, socio-economic and demographic factors and cultural and knowledge barriers on the demand side [26, 27]. Maternal health vouchers and other innovative financing initiatives have been employed in many impoverished settings in recent years and have successfully increased deliveries with skilled birth attendants and institutional deliveries [28–38]. In this paper, we report the effect of an integrated demand- and supply-side intervention undertaken as part of an ongoing maternal and newborn health improvement study on facility delivery rates over a 24-month period in a rural population in Sylhet district, Bangladesh.

## Methods

### Study design and setting

The Projahnmo study group, a research partnership of Johns Hopkins Bloomberg School of Public Health with the Bangladesh Ministry of Health and Family Welfare (MOHFW) and a number of Bangladeshi non-governmental organizations (NGOs), is participating in a study known as the Alliance for Maternal and Newborn Health Improvement (AMANHI) [39, 40]. One of the objectives of AMANHI is to establish a biorepository by collecting biological samples from pregnant women, their newborn babies and fathers of these babies with the initial aim of identifying hypothesized biomarkers of important adverse maternal and fetal outcomes. The biorepository study is being implemented in three countries including Bangladesh and is centrally coordinated by the World Health Organization (WHO). In Bangladesh, the study is being conducted in two rural sub-districts (Zakiganj and Kanaighat) of Sylhet district located in the northeastern part of the country. This region of Bangladesh has the lowest health indicators, including maternal care seeking, compared to other parts of the country [2].

The estimated population of the AMANHI study area is approximately 120,000. In the study area, the first level outpatient clinic in the public health sector, called Union Health and Family Welfare Centre (UH&FWC), which serves a population of approximately 25,000, with one clinic per union. A female paramedic (known as Family Welfare Visitor; FWV) in each UH&FWC provides maternal and child health services, including normal delivery. The FWV also conducts satellite clinics to provide family planning and antenatal and postnatal care. At the second tier of facility care, sub-district hospitals (*upazila health complex*, UHC) with both inpatient and outpatient facilities serve a population of approximately 250,000. A UHC is staffed by nurses, family welfare visitors, and physicians and provides both out-patient and in-patient services that include basic emergency obstetric care, normal delivery, caesarean sections, family planning, child immunization, and some other diagnostic and operative treatments.

The project started recruiting pregnant women in August 2014 who were permanent residents of the study areas and met the study eligibility criteria and then followed them up at 42 days after delivery. The study required collection of biological samples immediately after delivery, which is feasible only in facility deliveries. The project, therefore, designed a set of interventions to increase demand for and utilization of delivery care at health facilities within the study areas, namely, the Soroker Bazar UH&FWC, Manikpur UH&FWC and Zakiganj UHC. Approval for ethical conduct of human subject research was obtained from the Ethical Review Committee of the International Centre for Diarrhoeal Disease Research, Bangladesh (icddr,b) and the Institutional Review Board of the Johns Hopkins Bloomberg School of Public Health, USA. Administrative approval to conduct the study was obtained from Bangladesh Ministry of Health and Family Welfare. All the study participants provided written informed consent for collection of biological samples. Pregnancy and childbirth-related data were collected through standard pretested questionnaires. Statistical analysis was performed using Stata Version 13 (StataCorp LLC, Texas 77845 USA). Trend analyses for the facility delivery as a dichotomous variable were conducted using logistic regression.

### Intervention strategies

The study team devised an intervention package based on long standing experience in this community [41, 42] and started implementing the interventions in October 2014 with the assumption that a set of supply- and demand-side interventions can improve facility delivery rates in the study population.

#### Capacity expansion of the health facilities

Under the first strategy, between October and December 2014, the project made efforts to upgrade the designated health facilities by renovating the labor and delivery rooms, replacing the labor beds with new ones and by providing necessary equipment and commodities to all of the three health facilities.

#### 24/7 service coverage at the health facilities

The project placed six study nurses, after they had received their basic training on safe delivery and obstetric care, in the three designated health facilities in December 2014 in order to ensure 24/7 service coverage for obstetric care and to collect and process biological samples following a standard operating procedure.

#### Ensuring services by trained providers

With technical assistance and direct supervision from the Deputy Director of Family Planning of MOHFW, the project arranged a basic training program on safe delivery and emergency obstetric care for the FWVs working in the designated UH&FWCs as well as for the study nurses recruited by the project during November-December 2014.

#### Community advocacy meetings

Starting in December 2014, the project arranged a series of community advocacy meetings involving local religious scholars, community leaders, traditional birth attendants (TBA) and stakeholders and thereby attempted to build awareness, community support and social approval. These meetings were intended to sensitize the community and build support for facility delivery and other study activities. During the meetings, field staff, including the study physicians, discussed the local maternal and newborn health situation, raised awareness about the benefits of skilled birth attendance and maternal and newborn healthcare services, and provided information on the service availability in the designated health facilities.

#### Comprehensive birth planning counseling

In February 2015, the project made an approach to provide comprehensive birth planning counseling to the enrolled women in the presence of the senior members of the family, the family decision maker, neighbors, TBAs and village health workers (VHWs are field workers with a narrow range of tasks, and many of them are TBAs). During these sessions at the household level, the community health workers (CHWs) attempted to create awareness among the family members of the value of maternal health services and birth preparedness, provide information on sources of care, and encourage the pregnant women and families to avail facility delivery from designated health facilities. In addition, the field staff requested referrals from the TBAs since the families often rely on their opinions.

#### Cash incentives to cover expenses

In February 2015, the project undertook a cash incentive initiative for lowering financial barriers to accessing institutional delivery care. The project offered Taka 100 (US $1.3) to the families for active labor notification and Taka 1,500 (US $19.2) to the woman for giving birth in any of the designated health facilities (exchange rate in 2016: US $1 = Taka 78). A VHW received Taka 200 (US $2.6) and a TBA received Taka 500 (US $6.4) as incentive if she brings the mother to the health facility. Additionally, the project offered Taka 500 (US $6.4) cash incentive to the service provider at the health facility for attending each delivery of the study’s enrolled mothers.

#### Emergency transport and support of referral

The project deployed a full-time three-wheeler community vehicle, locally called the ‘CNG’, in February 2015 for the purpose of emergency transportation of the pregnant women from their respective households to the assigned health facilities. This ‘maternity vehicle’ seemed appropriate to the terrain and has demonstrated its ability to reach the doorsteps of the households of the pregnant women, even at night.

In addition to this, the project also offered financial supports during referral of complicated deliveries from the assigned health facilities to a tertiary care hospital (Sylhet Osmani Medical College Hospital; SOMCH) located in Sylhet in order to ensure proper care, including life-saving surgical interventions such as cesarean sections (C-section). In such referrals, the project provided assistance in arranging a road ambulance and reimbursed the families for the transport costs with a ceiling of Taka 1,000 (US $12.8).

#### Drugs and supplies

Starting in February 2015, the study nurses maintained a stock of essential drugs and surgical items for conducting a normal delivery at the assigned facilities. For any out-of-stock drugs that the family had to purchase from nearby shops/pharmacies, the project reimbursed the actual cost with a ceiling of Taka 1,000 (US $12.8) to the families.

## Results

**Fig 1:**
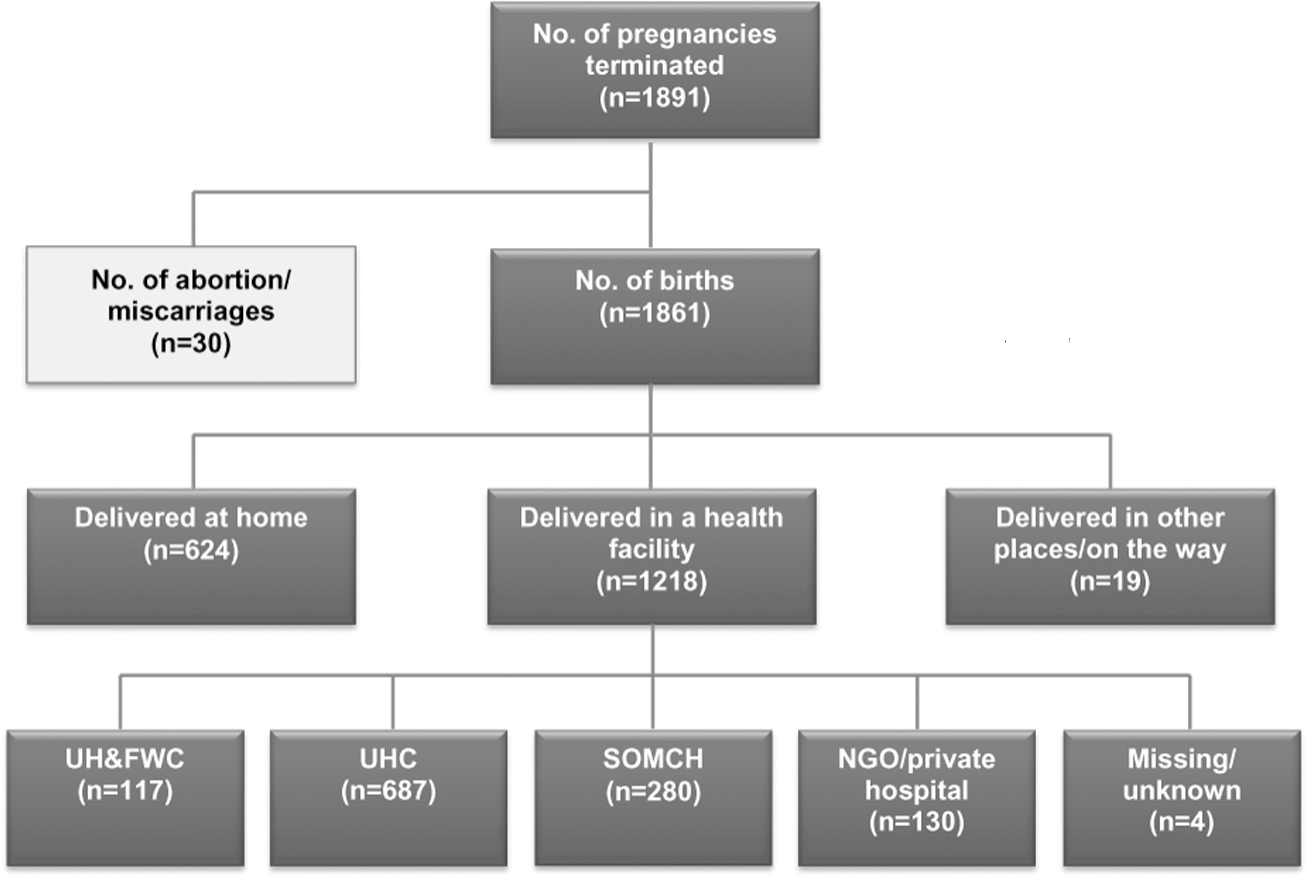
Study flow chart.

We present here the numbers of deliveries among women enrolled in the AMANHI biorepository study by place of delivery during the period of December 2014 to November 2016 (Fig 1). There was a statistically significant increase in the facility delivery rate over the study period (p < .0001). Thirty abortion cases, either spontaneous or induced, were excluded from the analysis. Overall, out of 1,861 deliveries, 624 deliveries (33.5 percent) occurred at home whereas 1,218 deliveries (65.4 percent) occurred in a health facility. Among the 1,218 institutional deliveries, the number of women who delivered in UH&FWCs was 117 (9.6 percent), in UHCs 687 (56.4 percent), in SOMCH 280 (23.0 percent) and in NGO/private hospitals130 (10.7 percent). Four deliveries (0.3 percent) were reported to taken place in a health facility but the names or types of the facilities are missing. In addition to these, there were 19 deliveries that occurred in places other than home or a health facility including on the way to a hospital.

**Fig 2:**
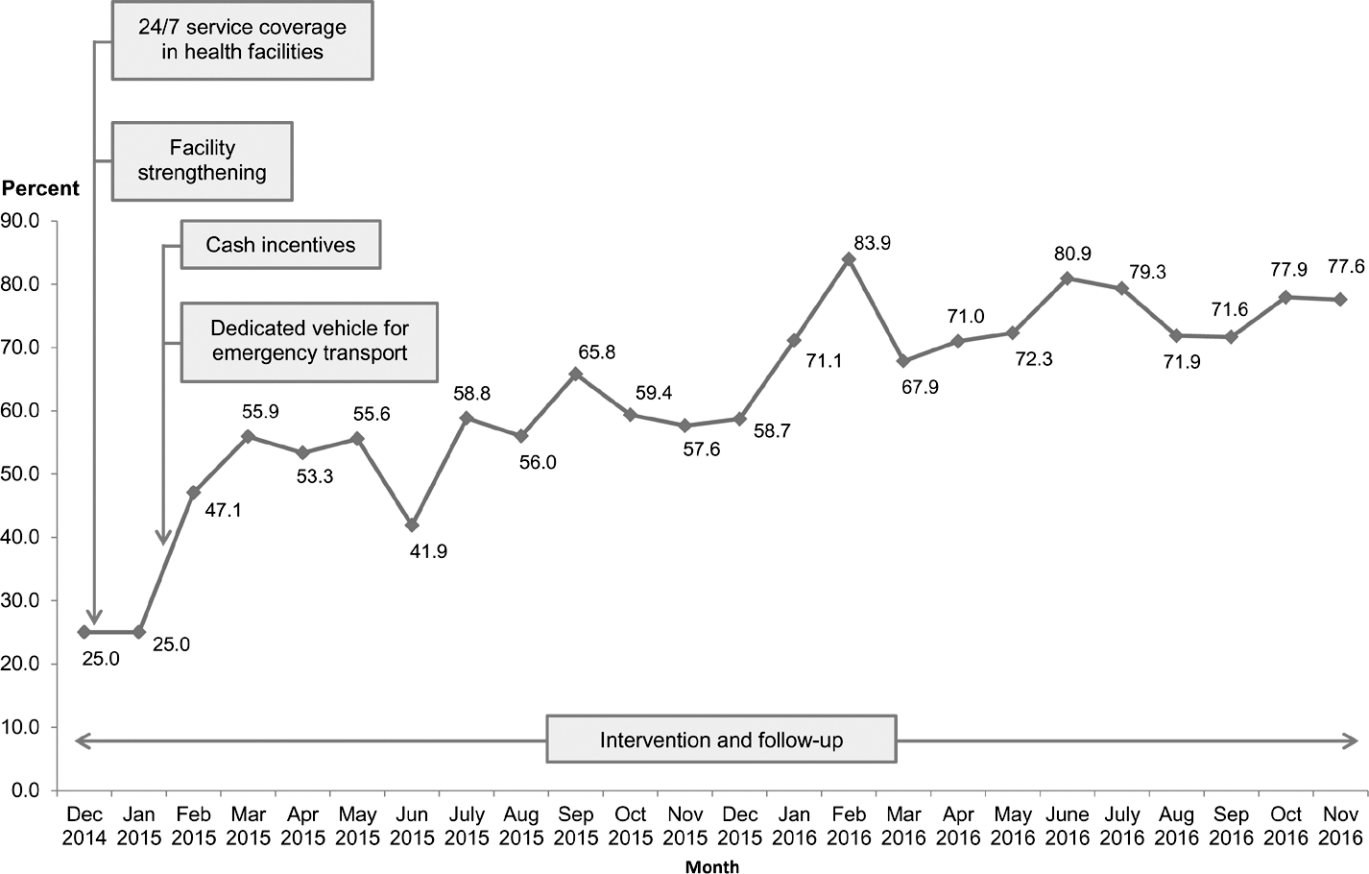
Trends in proportions of pregnant women delivered in a health facility.

The level of institutional delivery was quite low in the initial two months (25.0 percent in each of the months of December 2014 and January 2015) but rose to 47.1 percent in February 2015, which coincided with the start of the cash financial incentive initiative to cover expenses of accessing facility care for child birth (February 2015) as well as the deployment of a dedicated ‘maternity vehicle’ in the same month for transportation of pregnant women to the nearest health facility (Fig 2). The proportion of women delivering in a health facility increased further to 55.9 percent in March 2015 and then remained relatively stable up to December 2015 at approximately 55.0 percent, except for the month of Holy Ramadan (June-July 2015) when the response for health facility deliveries was relatively lower (41.9 percent).

An additional increase in the level of institutional delivery among women was observed in January 2016 with a 12.4 percentage point increase from that in December 2015 (71.1 percent vs. 58.7 percent). The maximum increase in the proportion of institutional delivery occurred in February 2016 (83.9 percent) and then remained steady at approximately 74.0 percent in the subsequent months. Overall, there was a 52.6 percentage point increase in the proportions of institutional deliveries between December 2014 (25.0 percent) and November 2016 (77.6 percent) in the study population.

## Discussion

In this population-based cohort of Bangladeshi women, the institutional delivery rate increased from 25.0 percent to 77.6 percent in 24 months. We observed that implementation of a set of integrated supply- and demand-side interventions was associated with a substantial increase in the utilization of health facilities for birthing care in a rural population of Bangladesh, which is consistent with the findings from other similar interventions including demand-side financing voucher programs in many other settings [27, 28, 32, 34, 36–38, 43–46]. We carried out this study in Kanaighat and Zakiganj sub-districts of Sylhet district, Bangladesh, and they were not included in the ongoing maternal health voucher scheme commonly known as the Demand-Side Financing (DSF) program, initiated by the Government of Bangladesh in 2007 [29–31]. To our knowledge, there were no other competing programs within our intervention areas that subsidized the cost of accessing services and can explain the changes that we have observed in our study.

The government of Bangladesh, since the 1980s, has been providing core services for maternal and child health for the rural population through district hospitals, UHCs, UH&FWCs, community-based skilled birth attendants (CSBAs) and community clinics. Guided by a number of global and national policies, plans and strategies, which have yet to realize their potentials, Bangladesh has primarily emphasized setting up health centers/hospitals, providing service training and upgrading equipment and supplies to improve the quality of health care [47]. To date, the financing of maternal health services in Bangladesh is largely supply based [29, 48], is not directly linked to the quantity of services, and does not consider the provider’s ability to reach the poorer sections of the community. At the community level, Family Welfare Assistants (FWAs) are responsible for delivering the domiciliary services that include providing ANC and referral of high-risk pregnancies alongside their family-planning tasks. However, although these workers are slated to make home visits every 2–3 months, only 20% of currently married women reported that they were visited by a fieldworker in the last 6 months [2]. A number of non-governmental organizations (NGOs) are playing a complementary role to the overall performance of the national maternal, child health and family planning (MCH-FP) program in Bangladesh. Nonetheless, they are mostly involved in the provision of primary healthcare at the grassroots level and do not adequately address barriers to access to appropriate obstetric care. Hence, despite significant improvements in the healthcare delivery infrastructure, access to maternal health services including facility-based delivery care in Bangladesh remained low [27, 47, 49].

The apparent failure of the supply-side strategy to reach the poor has prompted the governments, practitioners and funders in many countries to employ demand-side financing (DSF) including maternal health voucher schemes to encourage utilization of essential maternal health services. There have been emerging findings of increased uptake of institutional delivery or skilled attendance at birth associated with the DSF schemes from different parts of the world [28, 29, 32–38, 46]. In Bangladesh, the Ministry of Health and Family Welfare, with pooled funds from various sources and technical support from the WHO, started the implementation of the DSF maternal health voucher program in 33 sub-districts in 2007, which covered a population of approximately 10 million people [26, 27, 29–31]. The service components covered by the vouchers are transport costs, vouchers for antenatal care, safe delivery at home or in a facility, emergency care for complications, and postnatal care, plus a gift and cash incentive for delivery with a qualified provider. Women received up to Taka 500 (US $7.29) for routine transport costs, Taka 500 for emergency transport, a gift box worth Taka 500, and a Taka 2000 (US $29.18) cash incentive after delivering with a qualified provider (exchange rate in 2009: US $1 = Taka 68.55) [25, 29, 30]. In addition to this, the health care facilities as well as the providers received incentives for providing voucher-covered obstetric services at the following rates: Taka 300 (US $4.3) for a normal delivery, Taka 1000 (US $14) for forceps/vacuum, Taka 100 (US $1.4) for medicine, Taka 1000 (US $14) for management of eclampsia, and Taka 6000 (US $86) for a C-section. The average cost per voucher distributed (based upon the direct costs of the DSF program) is estimated to be US $41, which includes incentives to pregnant women as well as to providers and facilities in addition to the program administrative costs [31]. The DSF program in Bangladesh has shown that the women who received the intervention had an 18.8 percentage points increase in institutional deliveries (37.5 percent in DSF program sub-districts compared to 18.7 percent in control sub-districts) and 36.6 percentage points increase in use of a skilled provider during delivery (63.7 percent in DSF program sub-districts compared to 27.1 percent in control sub-districts) during the initial two years of the intervention [31].

Under a national conditional cash transfer (CCT)-based program known as Janani Suraksha Yojana (JSY) in India [32, 33, 45], all pregnant women in 18 high-focus states received a 1,400 rupee (US $28) cash incentive after delivery in a government or accredited private health facility. In addition, the community-level health workers received payments of 600 rupees (US $12) per delivery assisted by them. The JSY program reported a 42.6 percentage point increase in facility-based delivery after implementation [33]. A maternal health voucher scheme in Pakistan reported that purchase of a voucher booklet (valued at US$50; were sold for US$1.25 to low-income women targeted by project workers) containing redeemable coupons for three antenatal care visits, a postnatal care visit and institutional delivery was associated with a 22.1 percentage point increase in institutional delivery [43]. Poel et al. described an increased probability (10.1 percentage point increase) of having delivery in a public health-care facility following the implementation of a maternal voucher scheme in Cambodia [37]. A combined supply- and demand-side intervention in Uganda has demonstrated a substantial increase in the number of facility births (from <200 deliveries/month to over 500 deliveries/month) [36]. Bellows et al. found that allowing eligible women in Kenya to purchase vouchers that covered antenatal care, facility-based delivery, and postnatal care resulted in significantly greater odds of a facility-based delivery (OR: 1.4 (1.2–1.6)) among respondents, compared with similar respondents prior to voucher launch [38]. A similar result has been reported from rural Burkina Faso, with increased facility-based delivery rates from 49 to 84 percent during the intervention period when women were offered 80 percent subsidy for facility-based deliveries [46].

In our study, we observed a 52.6 percentage point increase in facility-based delivery after implementation of an integrated supply- and demand-side intervention over a 24 month period. The study is ongoing and the cost-effectiveness of the project, including average cost per institutional delivery, is yet to be evaluated; however, the total incentive we provided was comparable to the national program described above. In the first step of our intervention during October-December 2014, we made attempts to improve the quality and responsiveness of the service provision in three designated public health facilities. Upgrading and renovation of the health facilities were done as described earlier. Adding to this, the project deployed trained nurses and support staff to ensure 24/7 service coverage in all the designated health facilities. Nonetheless, despite the capacity expansion of the designated healthcare facilities, there were no visible improvements in the uptake of institutional birth among enrolled pregnant women until February 2015, when we addressed the financial barriers by introducing cash incentive payments to families and service providers. This may have reinforced the fact that supply-side interventions alone are not adequate to increase utilization of health care services in the low-utilization areas [5, 8, 50, 51], and financial barriers to access must be addressed.

In February 2015, the project emphasized the generation of demand among the women and their families, combined with lowering financial barriers to accessing institutional delivery care, through the arrangement of a series of advocacy meetings involving the community leaders and stakeholders, comprehensive birth planning counseling involving the family decision makers, the provision of cash incentives for giving birth in a health facility and the arrangement of emergency transport, including provision of a full-time dedicated ‘maternity vehicle’. Employment of this comprehensive demand-side intervention may have influenced the relative increase in the proportions of institutional deliveries in February 2015 (47.1 percent vs. 25.0 percent in January 2015), which have further increased in the following months. On the other hand, provision of a referral support for the complicated deliveries to a tertiary level hospital (i.e., SOMCH) was believed to help build a level of confidence and trustworthiness among the enrolled women and their families that may have enhanced the demand generation for delivering a child in any of the study-designated health facilities.

We have observed a steady trend in the proportions of institutional deliveries among enrolled women at approximately 53 percent throughout the year 2015, which rose to approximately 75 percent in the year 2016. A possible explanation for this increase could be that any new concerted effort to ameliorate maternal care seeking through supply- and demand-side interventions such as ours needs time to gain momentum, because it supposedly takes time for both the recipients and the service providers to become accustomed to the incentive scheme [27].

The intervention was novel and complex as well because it incorporated several activities in the community and intervened within the formal health care delivery system in a rural setting. It involved not only the family decision makers but also the traditional birth attendants, community resource persons and stakeholders and emphasized social approval and its ability to bring about changes in behavior. It targeted creating awareness of the value of maternal health services and providing information of sources of skilled obstetric care to the families. The project offered emergency transport available at the doorsteps of the rural households, which has gradually become popular and well-accepted among the recipients. It incorporated capacity expansion of healthcare facilities, allowing additional incentive payments to public health care providers, increased uptake through financial incentives to the households that reduce the cost of accessing services and developed effective referral systems for complicated cases to a tertiary care hospital.

The study had a number of limitations. This observational study within the context of an epidemiological study was based on a before-and-after design and did not have a concurrent control group. Therefore, it is harder to rule out the possibility that the observed increase in facility-based deliveries was influenced by the existence of another intervention or program or some other confounder. Another potential limitation is that we have recruited only a subset of pregnant women who met the study enrollment criteria in a relatively small total population of 120,000, which makes it difficult to draw confident conclusions about the generalizability of the intervention package. Moreover, in our study, we did not analyze the cost-effectiveness of the intervention, particularly the incremental cost per additional delivery in a public health facility that is required to facilitate health policy and decision making. We provided service training and upgraded equipment and supplies, but we do not have any additional data on quality of health care in the health facilities, which could be helpful. It is also difficult to determine which of the various strategies was more effective, because the intervention was integrated and provided as a package.

## Conclusions

This study provides evidence that integrated supply- and demand-side interventions addressing the access barriers can result in rapid increases in the utilization of facility-based services for maternity care in a resource-poor setting. Further improvements in maternal care seeking can be achieved if such a comprehensive approach explicitly addresses the transport difficulties in a rural context and gives ample consideration to the needs for a referral back-up for complicated deliveries. The study assembled various intervention designs and strategies that can be tailored to identify what combination of interventions may produce the optimum result. The tailored package needs to be implemented in a large-scale program using rigorous implementation research studies to draw confident conclusions on the impact of supply- and demand-side financing initiatives and provide information about their costs, feasibility and sustainability in order to make corresponding policy recommendations.

## Supporting information

S1 File Manuscript_Facility_Delivery_v4.4_12Aug2017_EDITED_AJE. (DOCX)
